# Retraction: Empirical analysis of the integration mechanism of explicit and implicit education on the ideological and political education and impact on mental health: the perspective of new media

**DOI:** 10.3389/fpubh.2023.1221658

**Published:** 2023-05-25

**Authors:** Frontiers Editorial Office

The journal retracts the article cited above.

Following publication, the publisher has found evidence of peer review manipulation. As the scientific integrity of the article cannot be guaranteed, and adhering to the recommendations of the Committee on Publication Ethics (COPE), the publisher therefore retracts the article.

This retraction was approved by the Chief Editors of Frontiers in Public Health and the Chief Executive Editor of Frontiers.

